# Differences in Characteristics Associated With Mobility in Older Dwellers of a Hillside Residential Community

**DOI:** 10.1093/geroni/igaa057.954

**Published:** 2020-12-16

**Authors:** Ryoichi Nitanai, Ryogo Ogino, Daisuke Umemoto, Yu Matsumura, Rika Sakurai, Kazutate Hosogaya, Takaaki Endo, Jun Goto

**Affiliations:** The University of Tokyo, Tokyo, Japan

## Abstract

In a super-ageing society, sustaining outings as an elderly person is necessary for maintaining good health and preventing frailty and social isolation. Avoiding driving cars and walking due to ageing are among factors that deter the elderly from going out. However, the characteristics associated with mobility on foot and by car in actual outings of the elderly have not been examined. To explore these characteristics, we conducted interviews with 23 elderly participants living in a suburban hillside residential community in Japan, and investigated the destinations, routes, and means of all their outings for a certain week. Then, spatial analysis was conducted to identify differences in behavioral characteristics associated with mobility on foot and by car/taxi among three age groups: 70–79, 80–84, and 85+. Consequently, two inclinations were identified. First, the older the group, the smaller the area of the outing on foot due to difficulties in walking on slopes for a long duration with luggage. Second, the use of a car/taxi varies among the three groups. While the 70–79 age group used cars/taxis for district-to-district trips, the 80–84 age group rarely used them, and further, the 85+ age group mainly travelled by them one-way or made back-and-forth trips. To summarize, for older suburban dwellers in a hillside residential community, mobility on foot is lowered by physical and mental weakness and the landscape. Therefore, even though mobility increases by using cars/taxis, willingness for outing has changed with ageing, and thus, they make limited use of opportunities.

